# Floral Color, Anthocyanin Synthesis Gene Expression and Control in Cape Erica Species

**DOI:** 10.3389/fpls.2019.01565

**Published:** 2019-11-28

**Authors:** N C Le Maitre, Michael David Pirie, Dirk U. Bellstedt

**Affiliations:** ^1^Bellstedt Laboratory, Department of Biochemistry, Stellenbosch University, Stellenbosch, South Africa; ^2^Johannes Gutenberg-Universität, Mainz, Mainz, Germany; ^3^University Museum, University of Bergen, Bergen, Norway

**Keywords:** floral color, anthocyanin, gene expression, *Erica*, RT-qPCR, UPLC-MS/MS

## Abstract

**Introduction:** The Cape Floristic Region (CFR) is a biodiversity hotspot, recognized globally for its unusually high levels of endemism. The origins of this biodiversity are a long-standing topic of research. The largest “Cape clade,” *Erica*, radiated dramatically in the CFR, its ca. 690 species arising within 10–15 Ma. Notable between- and within-species flower color variation in *Erica* may have contributed to the origins of species diversity through its impact on pollinator efficiency and specificity.

**Methods:** We investigate the expression and function of the genes of the anthocyanin biosynthesis pathway that controls floral color in 12 *Erica* species groups using RT-qPCR and UPLC-MS/MS.

**Results:** Shifts from ancestral pink- or red- to white- and/or yellow flowers were associated with independent losses of single pathway gene expression, abrogation of the entire pathway due to loss of the expression of a transcription factor or loss of function mutations in pathway genes.

**Discussion:** Striking floral color shifts are prevalent amongst the numerous species of Cape *Erica*. These results show independent origins of a palette of mutations leading to such shifts, revealing the diverse genetic basis for potentially rapid evolution of a speciation-relevant trait.

## Introduction

The Cape Floristic Region (CFR) at the southern tip of Africa is a globally recognized biodiversity hotspot (Mittermeier et al., 2004). It has levels of species richness not normally seen outside of the tropics, with ca.10,000 plant species of which ca. 7,000 are endemic to the region (Linder, 2003). Which factors have shaped and driven this spectacular radiation has been a long-standing question in evolutionary biology. The largest clade in the CFR is that of Cape *Erica* (Linder, 2003; Pirie et al., 2018). The ca. 690 species represent an endemic radiation (Pirie et al., 2016), with species of *Erica* found throughout the CFR, making it an ideal model system to study these forces.

The genus *Erica* (Ericaceae) is a group of acidophilic, woody shrubs. It originated in Europe (Mcguire and Kron, 2005; Mugrabi De Kuppler et al., 2015) and migrated southwards through Africa, colonizing the mountains of Central Africa, the Indian Ocean islands, the Drakensberg mountains of Southern Africa and the CFR at the southern tip of South Africa where colonization appears to have been followed by rapid radiation (Mcguire and Kron, 2005; Mugrabi De Kuppler et al., 2015; Pirie et al., 2016, Pirie et al., 2018). Most *Erica* species are found in the CFR, where ca. 690 of the ca. 865 species in the genus occur (Oliver, 1989; Oliver, 2000; Linder, 2003). Diversification rates, as well as ancestral areas and patterns of character evolution of *Erica* have been inferred based on phylogenies of nuclear and plastid DNA sequence markers representing up to around 60% of known species (Pirie et al., 2011; Mugrabi De Kuppler et al., 2015; Pirie et al., 2016). Speciation within the Cape clade was particularly rapid (Pirie et al., 2016), and this may be linked to the complex patterns of morphological variation and regional endemism displayed by *Erica* species and populations distributed across the mountainous landscape of the CFR. During the radiation of the Cape *Erica* clade, floral morphology and floral color shifted numerous times (Pirie et al., 2011; Van der Niet et al., 2014; Le Maitre, 2017). Shifts between distinct pollination syndromes (suites of characteristics that promote specific vectors such as different species of insects and birds), could have resulted in gene flow restrictions between populations ultimately driving speciation (Pirie et al., 2011). Floral color shifted from the plesiomorphic (ancestral) pink most common in the older, European, lineages (Mugrabi De Kuppler et al., 2015), that are primarily insect pollinated, to white, green, red, and yellow, (Le Maitre, 2017), notably in the Cape where these colors are associated with a greater diversity of pollinators, including sunbirds (Rebelo et al., 1985).

In angiosperms, floral color plays a major role in pollinator specificity and changes in color may result in pollinator shifts and pollinator mediated speciation (Waser, 1998; Rausher, 2008; Carlson and Holsinger, 2013; Van Der Niet et al., 2014). Flower colors are determined by three major classes of pigments: anthocyanin pigments formed by the anthocyanin biosynthesis pathway; (Mol et al., 1998; Grotewold, 2006), carotenoids; and betalains (reviewed in Tanaka et al., 2008). Crowden and Jarman (1976) analysed flower pigments in a number of *Erica* species using paper chromatography. They did not report carotenoids or betalains, but documented several different anthocyanins: pelargonidin-hexose, cyanidin-hexose, peonidin-hexose, delphinidin-hexose, petunidin-hexose and malvidin-hexose with cyanidin-hexose the most prevalent (Crowden and Jarman, 1976).

**Figure 1:**
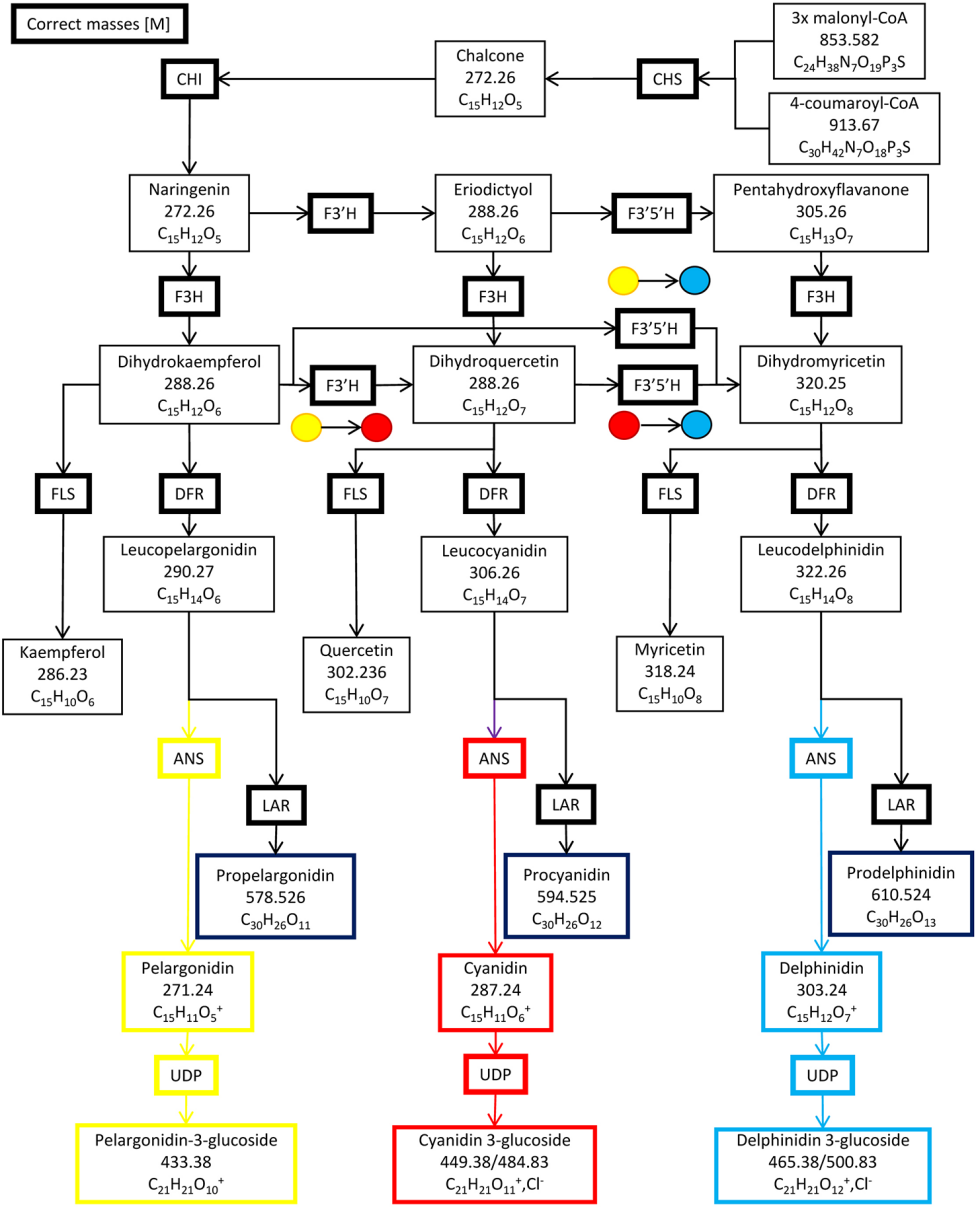
The anthocyanin biosynthesis pathway and side branches. Chalcone synthase (CHS), chalcone isomerase (CHI), flavanone hydroxylase (F3H), flavonoid 3′ hydroxylase (F3′H), flavonoid 3′, 5′ hydroxylase (F3′5′H) dihydroflavanol-4-reductase (DFR), anthocyanin synthase (ANS), UDP 3-O-gylcosyltransferase (UDP-GST), flavanol synthase (FLS) and leucoanthocyanidin reductase (LAR).

Six enzymes form the primary anthocyanin biosynthesis pathway (**Figure 1**), beginning with chalcone synthase (CHS), followed by chalcone isomerase (CHI), flavanone hydroxylase (F3H), dihydroflavanol-4-reductase (DFR), anthocyanin synthase (ANS) and lastly, UDP 3-O-gylcosyltransferase (UDP-GST) to synthesize pelargonidin 3-hexose. Two additional enzymes, flavonoid 3′ hydroxylase (F3′H) and flavonoid 3′, 5′ hydroxylase (F3′,5′H), can also further hydroxylate the product of F3H, resulting in the production of cyanidin 3-hexose and delphinidin 3-hexose respectively. Methylation of cyanidin–hexose produces peonidin–hexose, whilst methylation of delphinidin–hexose produces petunidin–hexose and/or malvidin–hexose. Intermediates in the pathway may be converted by flavanol synthase (FLS) or leucoanthocyanidin reductase (LAR) into flavanols or leucoanthocyanidins respectively (Wessinger and Rausher, 2012). The pathway is regulated by a complex of three transcription factors, WDR, MYB and bHLH in *Ipomoea purpurea*, and is conserved across angiosperms (Zhu et al., 2015). The complex of these three transcription factors binds to two highly conserved sites upstream of the 5′ UTR of each of the genes in the pathway, with MYB binding to the MYB recognition element (MRE) and bHLH binding to the bHLH recognition element (BRE), providing for unified control of the pathway (Zhu et al., 2015). Changes in floral color are due to loss of function mutations in enzyme(s) in the pathway; or loss of transcription factors by either loss of function mutations or loss of expression; or loss of transcription factor binding sites upstream of a gene (or genes) in the pathway through mutations (Wessinger and Rausher, 2012).

In this paper we describe genotypic changes potentially underlying the phenotypic changes in flower color in species broadly representative of the Cape *Erica* clade. Le Maitre et al. (2019) identified and sequenced the genes of the anthocyanin biosynthesis pathway and their *trans*-acting transcription factors in *Erica plukenetii* and developed a RT-qPCR assay for determining their expression levels. We use these techniques and UPLC MS/MS (Willemse et al., 2013; Willemse et al., 2014; Willemse et al., 2015; Stander et al., 2017) to test the hypothesis that changes in floral color are due to loss of function mutations in enzyme(s) in the pathway; or loss of transcription factors by either loss of function mutations or loss of expression; or loss of transcription factor binding sites upstream of a gene (or genes) in the pathway through mutations (Wessinger and Rausher, 2012). By analysing multiple examples of independent shifts in color in different clades in the genus we aim to link genotype to phenotype (floral anthocyanins and color) and identify general processes that may have played a role in driving the rapid diversification of *Erica* in the CFR.

## Materials and Methods

**Figure 2:**
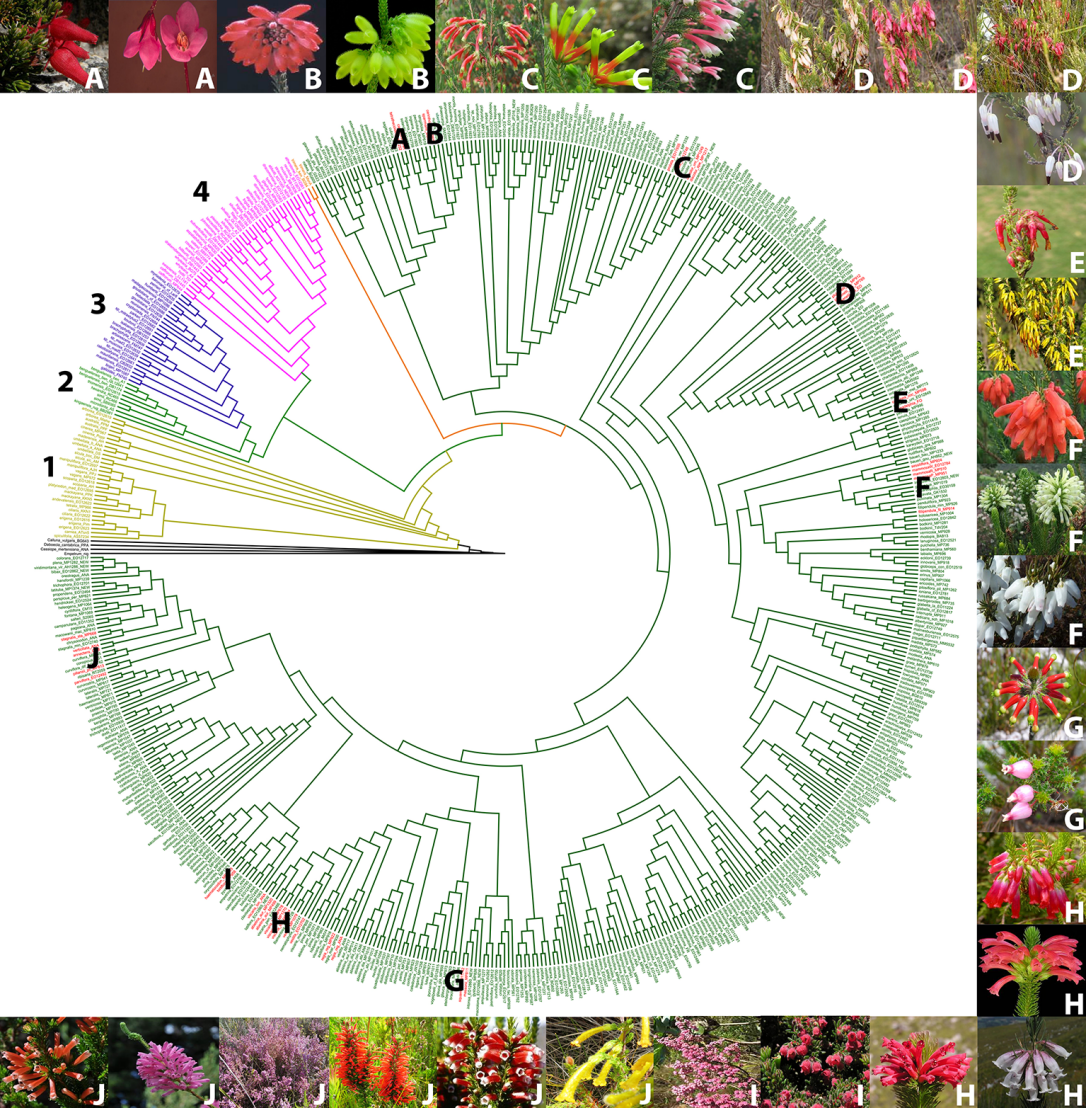
Phylogenetic tree of the genus *Erica*, with the major clades indicated with numbers, and the pink/red/white/yellow flowered species groups we investigated in the Cape clade indicated with letters (Le Maitre, 2017; Pirie et al., 2011, 2016). Outgroup species (black), 1. European clade (yellow), 2. African clade (light green), 3. Indian Ocean islands clade (purple), 4. Drakensberg clade (pink), *E. trimera* sister group to Cape Clade (orange) and Cape Clade (dark green). Red flowered species (red). **(A)** *E. cameronii* and *E. tetrathecoides* **(B)** *E. cerinthoides* and *E. sparrmannii* **(C)** *E. discolor ssp. speciosa, E. unicolor ssp. unicolor* and *E. prolata* **(D)** *Erica plukenetii* spp. *plukenetii* and *E. plukenetii* ssp. *breviflora,* **(E)** *E. monadelphia* and *E. coccinea* ssp. *coccinea* **(F)** *E. mammosa, E. sessiliflora* and *E. filipendula* ssp. *filipendula* **(G)** *E. massonii* and *E. squarrosa* **(H)** *E. regia* ssp. *regia, E. abietina* ssp. *abietina, E. viscaria* spp. *longifolia* and *E. vestita* **(I)** *E. haematocodon* and *E. hirtiflora* **(J)** *E. stagnais* ssp. *stagnalis, E. leucotrachela, E. pillansii, E. parviflora, E. verticillata* and *E. annectans.* Images of species are in the same order, clockwise from top left. Photo credits: South African National Biodiversity Institute, casabio.org (*E. tetrathecoides* and *E. massonii* ^©^ G Kirsten, *E. filipendula* ssp. *filipendula* ^©^ Cameron McMaster) and iNaturalist (*E. squarrosa* ^©^ Hayley-May Wittridge, *E. pillansii* and *E. stagnalis* ssp. *stagnalis* ^©^ Linkie).

Based on a phylogeny of *Erica* (Pirie et al., 2011; Pirie et al., 2016, Pirie ms. in prep) (**Figure 2**), we selected examples of color shifts from pink- to red-, white- or yellow flowered within the Cape *Erica* clade. These examples are distributed across the Cape *Erica* clade such that each would be expected to represent one or more independent color shifts. We analysed a total of 12 sets of closely related color variable species. We did not attempt to sample all members of monophyletic groups, nor strict sister-species pairs; instead for each example we selected a putatively plesiomorphic pink-flowered taxon and (a) closely related red-, and/or white- and/or yellow-flowered one(s) for investigation:

- *Erica cameronii* (red, bird) and *Erica tetrathecoides* (pink, insect)
- *Erica cerinthoides* (red, bird) and *Erica sparmannii* (white, insect)
- *Erica discolor* ssp. *speciosa* (red, bird), *Erica unicolor* ssp. *unicolor* (red, bird), and *Erica prolata* (pink, insect)
- *E. plukenetii* spp. *plukenetii* (red/pink/white, bird) and *E. plukenetii* ssp. *breviflora* (white, insect)
- *Erica monadelphia* (red, bird) and *Erica coccinea* ssp. *coccinea* (yellow, bird)
- *Erica mammosa* (red, bird), *Erica sessiliflora* (white, bird), and *Erica filipendula* ssp. *filipendula* (white, insect)
- *Erica massonii* (red, bird) and *Erica squarrosa* (pink, insect)
- *Erica regia* ssp. *regia* (red, bird), *Erica abietina* ssp. *abietina* (red, bird), *Erica viscaria* spp. *longifolia* (white, insect), and *Erica vestita* (red, bird)
- *Erica haematocodon* (red, insect) and *Erica hirtiflora* (pink, insect)
- *Erica stagnalis* ssp. stagnalis (yellow, insect), *Erica leucotrachela* (red, bird), *Erica pillansii* (red, bird), *Erica parviflora* (pink, insect), *Erica verticillata* (pink, bird), and *E. annectans* (red, bird)

All specimens were collected in the CFR. Identifications were confirmed by Dr E.G.H. Oliver, the longstanding taxonomic expert on the genus. Pollinator syndromes from Rebelo et al. (1985).

The analyses were restricted to the anthocyanin biosynthesis pathway. Other pigments, notably carotenoids (Tanaka et al., 2008) can also influence flower color, but have not yet been found in species of *Erica* (Crowden and Jarman, 1976; Ojeda et al., 2019), and we did not further test for their presence. Primer pairs were designed to bind to the conserved exonic regions of the genes of the anthocyanin biosynthesis pathway (Le Maitre, 2017). Using the methodology of Le Maitre et al. (2019), expression of the genes of the anthocyanin biosynthesis pathway and its *trans*-acting transcription factors at three different growth points of the corolla in each species was evaluated. The Qiagen Total RNA Qiagen RNeasy Power Plant Kit with the Qiagen RNase-Free DNase Set was used to extract total RNA from 50 mg of corolla tissue. Gel electrophoresis was used to check RNA quality. To confirm that these samples contained high quality RNA their 260/280 absorbance ratios were determined using a Nanodrop ND-1000 spectrophotometer. For the RT-qPCRs, the RT-qPCR mix was prepared using the Kapa SYBR Fast Universal Kit as follows: “10 µl PCR Buffer; 0.4 µl 10 mM Forward and 0.4 µl 10 mM Reverse Primer (**Table 1**); 0.4 µl 50X Reverse Transcriptase; 2 µl extracted RNA and 6.8 µl milliQ H_2_O to make up a total of 20 µl. RT-qPCR was performed in a Roche LightCycler 96, with two preincubation steps of 61°C for 5 min, then 95°C for 30 s, followed by 50 cycles of 95°C for 3 s, 58°C for 10 s and 72°C for 2 s. A melting step followed with 95°C for 10 s, 65°C for 1 min and 95°C for 1 s, verifying the presence of a single amplicon per reaction” (Le Maitre et al., 2019).

**Table 1:** The primers used for RT-qPCR of the genes of the anthocyanin biosynthesis pathway and its transcription factors (Le Maitre et al., 2019).

Name	Sequence	Expected product size (bp)
ANS-1,281F	AGTCCTCTCCCTAGGCTTGG	167
ANS-1,448R	ATGAAGGTGAGGGCGCTTAC	
CHI-632F	ACGGGCAAGCAATACTCAGA	184
CHI-816R	CTAACCGTTAGCGACCCCAG	
CHS-153F	CCGTCATGGCTATCGGGAC	109
CHS-262R	CTCCTTCAACTCGGCCTTGT	
DFR-112F	AGGATAACGTGAACGGCTCG	117
DFR-229R	ACGGTGGCTCGAACAACATA	
F3′5′H-261F	CGGAGATGCTCACGTACTCC	139
F3′5′H-400R	GTTGAATAAACCGGCCGACG	
F3′H-406F	CTCCGGGGCCAAGCATATT	125
F3′H-531R	AAGTCGTCCAAGGCCTTAGC	
F3H-259F	GATATCGCTAGCCGGGATCG	127
F3H-386R	TAATCAGACCGGCATCCACG	
UDP-GST-480F	AAGTCGCCGCAAAGTTCAAC	135
UDP-GST-615R	GGCACCAAAAAGGGTTCGTC	
PTB1-1,416F	TTCATCAGAACCGGCTCAGG	148
PTB1-1,564R	TGCTGACAAGACGTGCATCA	
MYB-998F	ATAACCCAAAGCCCACGAGG	136
MYB-1,134R	CACCCGATCAACCTCAGCTT	
bHLH-645F	AGTTGCGGAGGGATAGGCTA	151
bHLH-796R	GTCTGTTCTGGGAGGCCTTC	
WDR-1,605F	CAGGACCCCAGGTATACGGA	151
WDR-1,756R	CCTCACTCGCACTGTGGAAT	

For each taxon (species or subspecies) assessed, expression of each gene of the anthocyanin biosynthesis pathway was analysed with three biological replicates (flowers of very similar age from the same plant) of juvenile, intermediate and adult flowers, with two technical replicates of each. These groups of reactions per gene (and technical duplicates) included both no-reverse transcriptase and no-template controls. Numerous different primer pair combinations for each gene were tested to identify those that gave consistent amplification across all species. For this subset of consistently performing primers pairs, optimal primer concentrations and annealing temperatures were compared in order to select a final subset of primer pairs and identify a universal set of conditions that would deliver similar quantification cycle values (Cq), amplification efficiencies, and slopes when analysed in a single 96 well plate. Presence of a single amplicon was verified using melting curve analysis (Le Maitre et al., 2019). The reference gene, Ep-PBT1, was used to normalise the expression of the genes of the anthocyanin biosynthesis pathway, using the Roche LightCycler 96 software.

UPLC-MS/MS analysis was performed to evaluate differences in anthocyanin pigment production in each species. Corolla tube tissue (200 mg) was immersed in a 500 µl methanol, 1% formic acid, solution for 1 h in a sonicator and then centrifuged briefly in a low speed benchtop Eppendorf tube centrifuge. Supernatants were loaded on a Waters Synapt G2 instrument for UPLC analysis. The compounds were run in ESI negative on a Waters T3, HH, 2.1×150 mm column using a linear 0.1% formic acid (A) to acetonitrile gradient (B) at a flow rate of 0.250 ml/min. Initial conditions were 0% B and the conditions were kept constant for 1 min. The gradient was increased to 28% B at 22 min, with a subsequent increase to 40% B at 22.5 min. After a further increase to 100% B at 23 min, conditions were kept isocratic for 1.5 min. Conditions were changed back to the initial conditions and were kept constant for 4.5 min to wash the column. The 29 min run was monitored with a tandem mass spectrometer. Two biological replicates were used for each species analysed. The tandem mass spectrometer data were acquired using identical methodology and parameters as described by Willemse et al. (2014) and collected using MassLynx v4.1 software (Waters).

Where the RT-qPCR expression analysis implicated loss of expression of genes and/or UPLC-MS/MS analysis implicated loss of function mutations, in floral color shifts, those genes and/or their upstream regulatory regions were amplified by PCR using primers derived from the Next Generation Sequencing data acquired during the partial sequencing of the *E. plukenetii* genome (Le Maitre et al., 2019) and sequenced using the BigDye Terminator v3.1 cycle sequencing kit (Thermo Fischer Scientific) and the STeP sequencing protocol (Platt et al., 2007) in an Applied Biosystems Veriti thermal cycler. Sequencing electrophoresis was performed at the Central Analytical Facility, Stellenbosch University.

## Results

**Figure 3:**
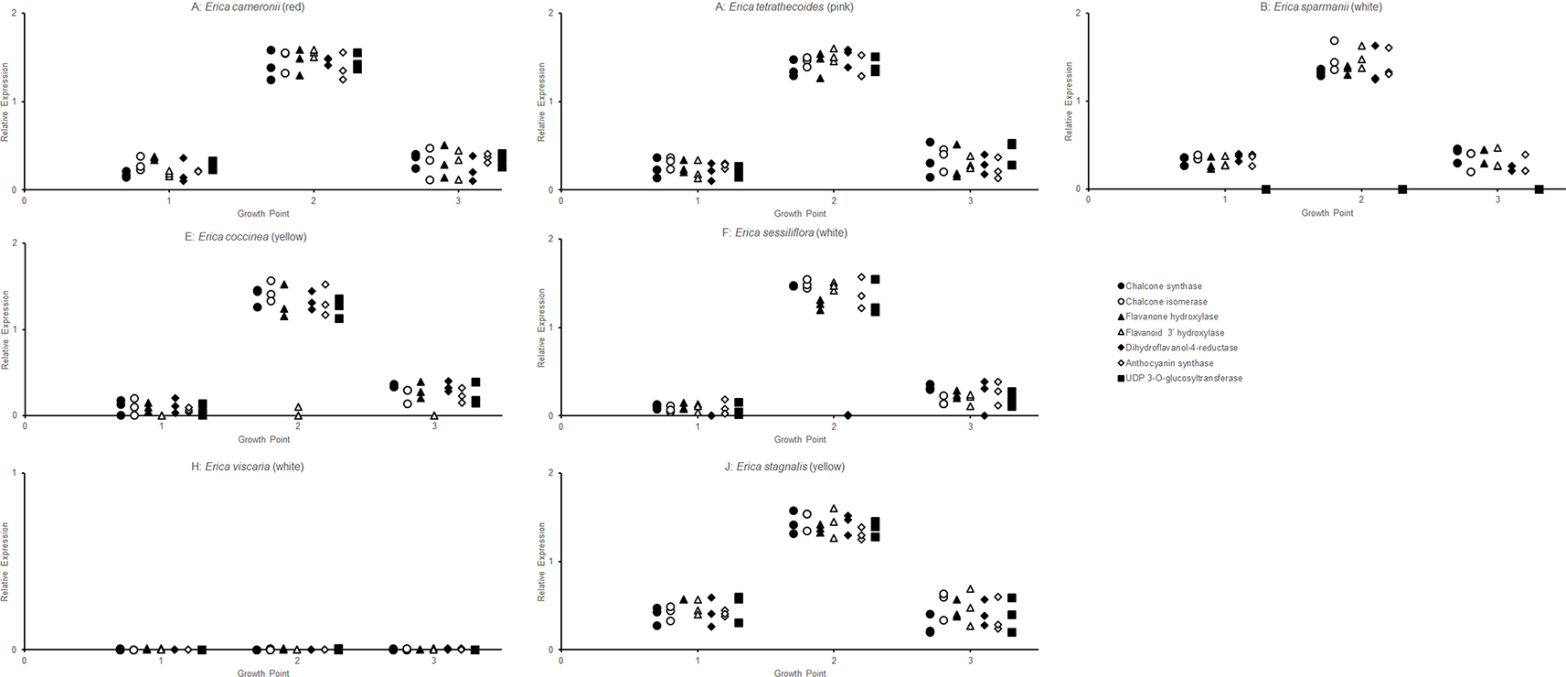
RT-qPCR analysis of the expression of the genes of the anthocyanin biosynthesis at three different growth points of the corolla in each species was evaluated, with three biological replicates of each growth point.

**Figure 4:**
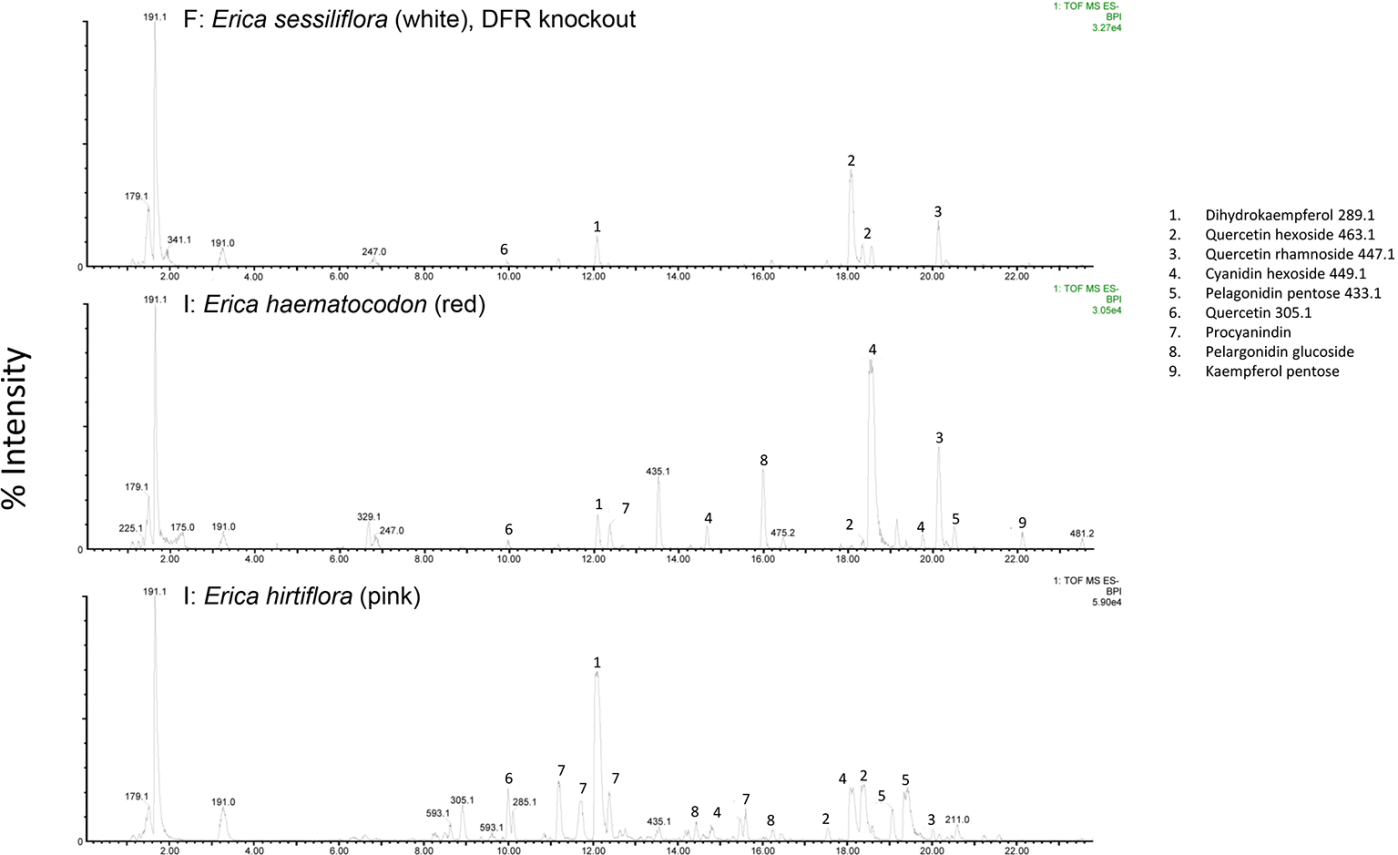
UPLC-MS/MS results from white-, red- and pink flowered species. Anthocyanin biosynthesis pathway products and intermediates are indicated (1-9). Dihydroflavanol-4-reductase (DFR) was knocked out in the white flowered species.

The RT-qPCR methodology from Le Maitre et al. (2019) originally developed for *E. plukenetii* was successfully used in multiple *Erica* species (**Figure 3**, **Table 2** and **Supplementary Figure 1**). The anthocyanins and the intermediates in the anthocyanin biosynthesis pathway were successfully detected using UPLC-MS/MS in each of the species tested (**Figure 4** and **Supplementary Figure 2**). Using these results, we followed a logical path for determining the lack of production of an anthocyanin precursor or product in a species or subspecies. Firstly, we assessed expression levels of the anthocyanin synthesis genes or transcription factors. Loss of expression of ANS, F3′H and DFR due to mutations in the upstream promotor binding region in *E. plukenetii* ssp. *plukenetii, E. coccinea* and *E. sessiliflora* respectively and mutations in the upstream promotor binding region of UDP-GST in both *E. plukenetii* ssp. *breviflora* and *E. sparmannii*, and a complete knockdown of all anthocyanin biosynthesis pathway expression due to loss of bHLH transcription factor expression in *E. viscaria* were detected using RT-qPCR (**Table 2**). In these instances, abrogation of transcription factor binding was the reason for the loss of anthocyanin intermediates or anthocyanins per se. Secondly, where gene expression analyses could not explain the floral color shifts, the non-functional enzymes, DFR and F3′H, were identified by the presence of their precursors and absence of their products in the UPLC-MS/MS analysis in *E. filipendula* and *E. stagnalis* respectively. This was confirmed by sequencing of their respective genes, which led to the identification of the loss of much of exon 2 of DFR in *E. filipendula* and a frameshift mutation in exon 1 of F3′H in *E. stagnalis.* Normal production of anthocyanin pigments was shifted to proanthocyanidins in *E. filipendula* and pelargonidin-hexoses in *E. stagnalis* (**Table 2**), thereby confirming the non-functionality of the respective enzymes that would lead to their formation. In *E. sessiliflora*, which possessed a defective transcription factor binding motif of the DFR gene, and in *E. filipendula*, which possessed a mutation in the DFR gene itself, the metabolites were channelled into kaempherol and quercetin, in both cases. Shifts between pink- and red flowers could not be explained by differences in gene expression profiles (**Figure 3**). Comparing the UPLC-MS/MS data from pink- and red flowers did not show any qualitative differences in the anthocyanins produced (**Figure 4**). There were some differences in non-anthocyanin related compounds but these are unlikely to determine to the final floral color.

**Table 2:** The results of the RT-qPCR and UPLC-MS/MS testing on the *Erica* species that were collected (Le Maitre et al., 2019). MYB recognition element (MRE).

Group	Species	Color	Gene expression	Anthocyanin production	Putative cause
A	*cameronii*	Red	Normal	Normal	No difference determined to wt
A	*tetrathecoides*	Pink	Normal	Normal	Wildtype
B	*cerinthoides*	Red	Normal	Normal	Wildtype
B	*sparmannii*	White	UDP-GST not expressed	Very little anthocyanins	No MRE
C	*discolor*	Red	Normal	Normal	No difference determined to wt
C	*unicolor*	Red	Normal	Normal	No difference determined to wt
C	*prolata*	Pink	Normal	Normal	Wildtype
D	*plukenetii plukenetii*	White	ANS not expressed	No intermediates after ans, procyanidins	2bp sub in MRE
D	*plukenetii plukenetii*	Pink	Normal	Normal	Wildtype
D	*plukenetii plukenetii*	Red	Normal	Normal	No difference determined to wt
D	*plukenetii breviflora*	White	UDP-GST not expressed	Very little anthocyanins	C to A mutation in MRE
E	*monadelphia*	Red	normal	Normal	Wildtype
E	*coccinea*	Yellow	F3′H not expressed	Perlagonidin-hexose, not cyanidin	No MRE
F	*mammosa*	Red	Normal	Normal	Wildtype
F	*sessiliflora*	White	DFR not expressed	No intermediates after dfr, kaempferol/quercetin	Large ∼500 bp insertion in promoter region
F	*filipendula*	White	Normal	No intermediates after dfr, kaempferol/quercetin	Missing most of exon 2
G	*massonii*	Red	Normal	Normal	No difference determined to wt
G	*squarrosa*	Pink	Normal	Normal	Wildtype
H	*regia*	Red	Normal	Normal	Wildtype
H	*abietina*	Red	Normal	Normal	Wildtype
H	*viscaria*	White	bHLH not expressed	No intermediates, except quercetins	bHLH not expressed
H	*vestita*	Red	Normal	Normal	Wildtype
I	*haematocodon*	Red	Normal	Normal	No difference determined to wt
I	*hirtiflora*	Pink	Normal	Normal	Wildtype
J	*stagnalis*	Yellow	Normal	Perlagonidin-hexose, not cyanidin	Frameshift mutation in exon 1 of F3′H
J	*leucotrachela*	Red	Normal	Normal	No difference determined to wt
J	*pillansii*	Red	Normal	Normal	No difference determined to wt
J	*parviflora*	Pink	Normal	Normal	Wildtype
J	*verticillata*	Pink	Normal	Normal	Wildtype
J	*annectans*	Red	Normal	Normal	No difference determined to wt

Shifts from red- or pink- to yellow flowered such as in *E. stagnalis* and *E. coccinea* were associated with flux shifts through the pathway, producing pelargonidin-hexose rather than cyanidin-hexose. In cases where enzymes in the pathway were either not present or non-functional such as *E. plukenetii* ssp. *plukenetii*, *E. plukenetii* ssp. *breviflora*, *E. sparrmanii* and *E. sessiliflora*, flux was diverted to other end products such as the leucoanthocyanidins, flavanols or procyanidins. Where the entire pathway was downregulated due to the loss of a transcription factor such as in *E. viscaria*, some central metabolites, such as quercetin were still found, probably produced by catabolism of other compounds.

## Discussion

Our approach, using RT-qPCR and UPLC-MS/MS, was successful in determining the probable causes of the floral color shifts between red- and/or pink- and white- or yellow flowered species. RT-qPCR and UPLC-MS/MS could not distinguish between pink- and red flowered species, likely due a combination of factors: it may well be that the shift between pink- and red flowers is due to a minor shift in anthocyanin intensity (Grotewold, 2006), a modification of the anthocyanin pigment with covalent metal ions (Akbari et al., 2012; Akbari et al., 2013), changes in vacuolar pH (Akbari et al., 2012; Akbari et al., 2013) or even cell shape (Kay et al., 1981; Noda et al., 1994; Bailes and Glover, 2018), or even the influence of other pigments, such as carotenoids (Tanaka et al., 2008), that might yet be detected in species of *Erica*.

Numerous mutations were identified as probable causes of floral color shifts in the *Erica* species studied. These covered the full spectrum of potential causes (Wessinger and Rausher, 2012) from loss of single gene expression due to transcription factor binding site mutations, loss of transcription factor expression, or functional mutations in a gene resulting in flux shifts between legs of the pathway. The wide variety of mutations associated with the color shifts supports the independent origins of the floral color shifts from the plesiomorphic pink form that had been identified using phylogenetic inference (Pirie et al., 2011; Pirie et al., 2016).

A single study had previously used paper chromatography to identify anthocyanins as the pigments that color flowers in *Erica* species (Crowden and Jarman, 1976). Ours is the first study to make use of UPLC-MS/MS. Six of the 30 species assessed by Crowden and Jarman (1976) were also analysed here: *E. discolor, E. plukenetii, E. coccinea, E. mammosa, E. regia* and *E. vestita.* However, Crowden and Jarman (1976) did not report the colors or the subspecies for the flowers they used, which makes comparison of the results problematic. Insofar as the results can be directly compared, i.e. with regard presence of particular anthocyanins in *E. mammosa* and *E. vestita,* they are consistent. However, our results go beyond those of Crowden and Jarman (1976), showing that the production of anthocyanins, by the anthocyanin biosynthesis pathway in *Erica*, appears to be identical to that of other angiosperms (Wessinger and Rausher, 2012; Zhu et al., 2015). Ojeda et al. (2019) studied *E. coccinea* ssp. *coccinea* using HPLC and their results correspond with ours, with red flowers due to anthocyanin production and its loss associated with floral color shifts.

Floral color shifts are associated with pollinator shifts and consequent reproductive isolation and diversification/speciation (Rausher, 2008; Wessinger and Rausher, 2012). Pollinator shifts are also associated with changes in a suite of characteristics relevant to pollinator selection. These include, but are not limited to, floral color, shape, size, scent and position, branch strength, growth form and habitat (Fenster et al., 2004; Van der Niet et al., 2014). Pollinator shifts are therefore the result of an accumulation of changes that may gradually change the optimal pollinator type from one to another. The color shifts that we have investigated are an important part of these shifts between different biotic pollinators and from biotic- to wind-pollination (Wolowski and Freitas, 2015).

The shifts from pink- to red flowers in Cape *Erica* likely represent adaptation to bird pollinators that are not found in the genus’ ancestral European range. These shifts are associated with other changes in a suite of characteristics that make the plants more suited to bird pollination, such as sturdier branches more suitable for perching; longer, more curved floral tubes that better mimic the beaks of local nectarivores such as *Anthobaphes violacea* (Orange Breasted sunbird), *Nectarinia famosa* (Malachite sunbird), *Cinnyris chalybeus* (Southern double-collared sunbird) and *Cinnyris afer* (Greater double-collared sunbird) (Rebelo et al., 1985; Rebelo and Siegfried, 1985; Van der Niet et al., 2014). In *E. plukenetii* ssp. *breviflora*, which has reverted to insect pollination, loss of anthocyanin production and the consequent shifts from red- or pink- to white flowered are associated with branches becoming less sturdy, reduction of floral tube length and flowers producing a distinct scent, promoting pollination by short-tongued noctuid moths (Van der Niet et al., 2014), rather than sunbirds. In other species of *Erica*, distinct combinations of multiple morphological traits (“syndromes“) are linked to pollination by different guilds of insects, including both generalist pollinators such as bees and specialists such as long proboscid flies; wind pollination; or even pollination by mammals (Rebelo et al., 1985; Rebelo and Siegfried, 1985; Turner et al., 2011; Lombardi et al., 2013; Bouman et al., 2017). Both the color shifts that we have investigated, and the associated changes in other characteristics, may have contributed to a niche occupation and specialization process, potentially in sympatry, and have therefore played a role in driving the diversification of *Erica* within the CFR.

Previous studies of anthocyanin synthesis and the genes regulating its production have focussed on color variants in a single species (Rausher and Fry, 1993; Coberly and Rausher, 2003; Jin et al., 2016) or a number of variants in closely related species (Forest et al., 2014; Ng and Smith, 2016) and thus general information across all angiosperms could be accumulated. The high species richness of *Erica* and the independent shifts in flower color apparent given the phylogeny, have enabled us to investigate the different mechanisms controlling anthocyanin synthesis within a single clade of closely related species. In accordance with our hypotheses, we have found evidence that mutations in transcription factor binding sites and functional genes as well as abrogation of the expression of a transcription factor were implicated in color shifts. An analysis of the frequency of these mutational changes shows that five can be attributed to transcription factor binding site mutations, two can be attributed to the mutation of functional genes and only one can be attributed to loss of transcription factor expression i.e. bHLH. This gives support to the evo devo hypothesis of Carrol et al. (2005), which has been extensively debated by Hoekstra and Coyne (2007), that: *cis*-regulatory changes are more likely to occur as they are thought to be relatively free of negative pleiotropic effects on fitness; mutational changes to functional genes are less likely because they are will have greater effects; and mutational changes to transcription factors genes are the least likely due to potentially more wide-reaching effects. In no instances were identical mechanisms found across species. This is to be expected, given independent origins and the stochastic nature of the evolutionary process, but is nevertheless striking when considered in the context of the multiple changes in flower color between and within the numerous closely related species in *Erica*. It points to a highly dynamic system allowing rapid adaptation to specific pollinators.

## Data Availability Statement

The gene expression data generated can be found in the SUNScholarData repository under https://scholardata.sun.ac.za/s/8eb50fa0cf88c0000ed0.

## Author Contributions

NL and DB collected samples and designed the study. MP provided phylogenetic data. NL performed the experiments and conducted the data analyses. NL , DB, and MP wrote the manuscript.

## Funding

This work was supported by the South African National Research Foundation (grant number 98867). MP was supported by the Heisenberg Program of the Deutsche Forschungsgemeinschaft (PI 1169/3-1).

## Conflict of Interest

The authors declare that the research was conducted in the absence of any commercial or financial relationships that could be construed as a potential conflict of interest.

## Supplementary Material

Supplementary Figure 1 The expression of the genes of the anthocyanin biosynthesis pathway as determined by RT-qPCR for all of the species analysed in the study.

Supplementary Figure 2 The UPLC-MS/MS for all of the species analysed in the study.
