# Supplementary figures and images for: Floral Color, Anthocyanin Synthesis Gene Expression and Control in Cape Erica Species
Source: Front Plant Sci. 2019 Nov 28;10:1565. doi: 10.3389/fpls.2019.01565 (PMC6892755; doi:10.3389/fpls.2019.01565)

# RT-qPCR results for all *Erica* species tested

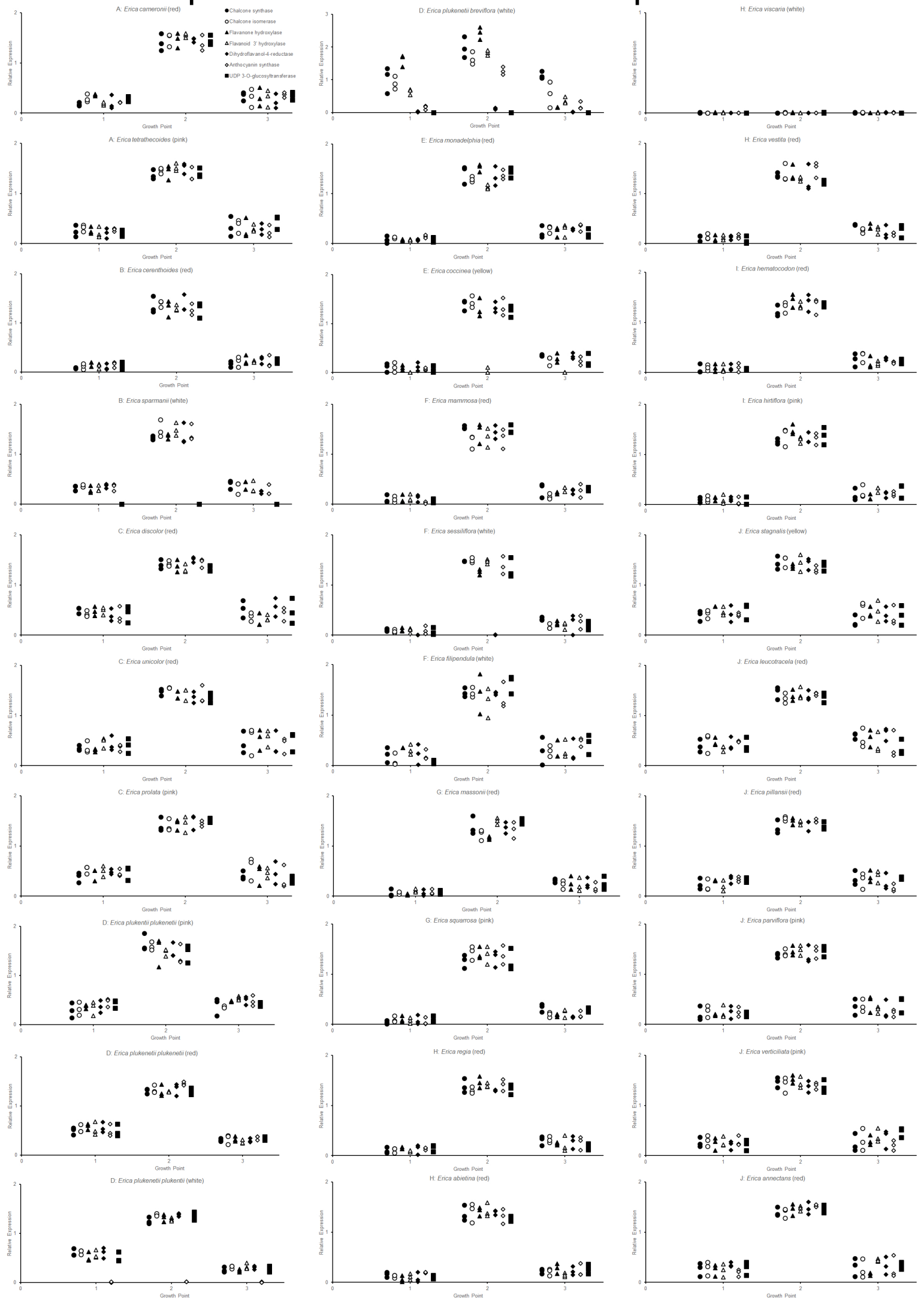

Supplement: Supplementary Figure 1 — The expression of the genes of the anthocyanin biosynthesis pathway as determined by RT-qPCR for all of the species analysed in the study. [file DataSheet_1.pdf]
