# Supplementary material for: Floral Color, Anthocyanin Synthesis Gene Expression and Control in Cape Erica Species
Source: Front Plant Sci. 2019 Nov 28;10:1565. doi: 10.3389/fpls.2019.01565 (PMC6892755; doi:10.3389/fpls.2019.01565)
Supplement: Supplementary Figure 2 — The UPLC-MS/MS for all of the species analysed in the study. [file DataSheet_2.pdf]

Results of the UPLC-MS/MS analyses on *Erica* sp. floral tissue of various colours

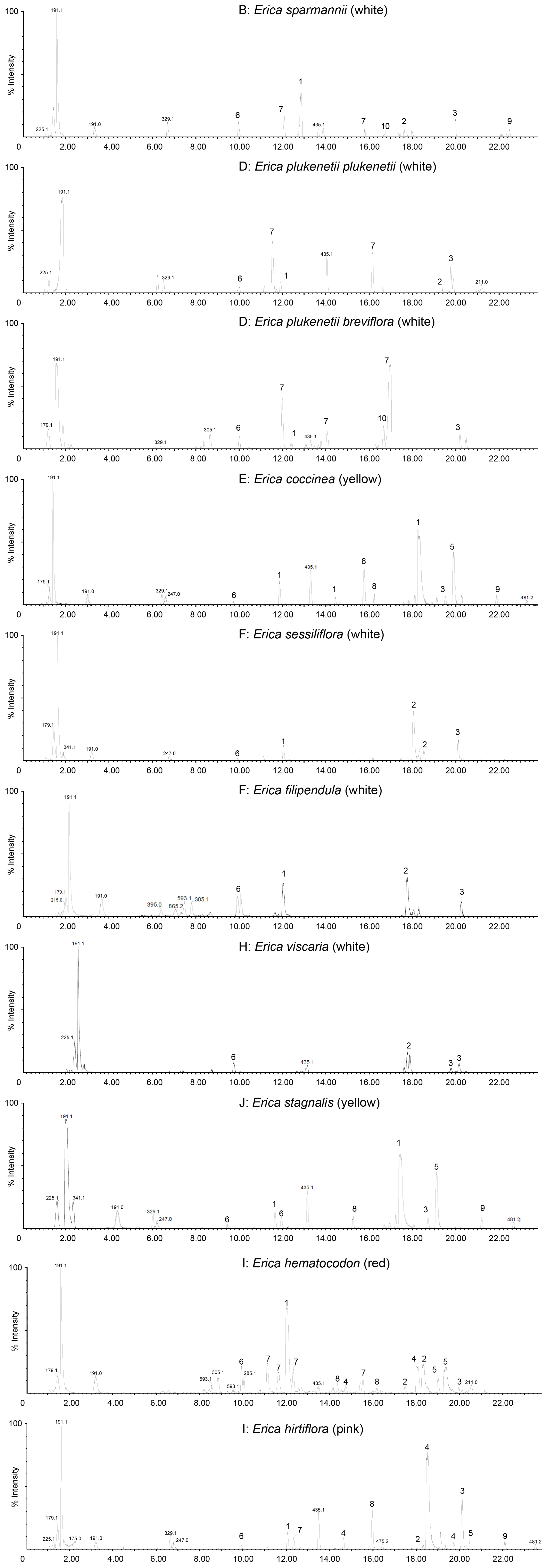

1.) Dihydrokaempferol 289.1
2.) Quercetin hexoside 463.1
3.) Quercetin rhamnoside 447.1
4.) Cyanidin hexoside 449.1
5.) Pelagonidin pentose 433.1
6.) Quercetin 305.1
7.) Procyanindin
8.) Pelargonidin glucoside
9.) Kaempferol pentose
10.) Cyanidin
